# Differences in characteristics of pediatric patients undergoing computed tomography between hospitals and primary care settings: implications for assessing cancer follow-up studies

**DOI:** 10.1186/s13584-015-0031-x

**Published:** 2015-11-15

**Authors:** Gabriel Chodick, Moran Levin, Ruth A. Kleinerman, Michael Shwarz, Varda Shalev, Shai Ashkenazi, Gad Horev

**Affiliations:** Medical Division, Maccabi Healthcare Services, Tel Aviv, Israel; Division of Cancer Epidemiology and Genetics, National Cancer Institute, Bethesda, MD USA; Sackler Faculty of Medicine, Tel Aviv University, Tel Aviv, Israel; Schneider Children’s Medical Center in Israel, Petach-Tiqva, Israel

**Keywords:** Computed tomography, Pediatrics, Hospitalization, Israel

## Abstract

**Background:**

Recently published analyses showed that computed tomography (CT) scans in pediatric patients are associated with increased risk of radiation-related cancer. These analyses were based on data collected both from either hospitals and primary care services. Study objectives were to characterize cohorts of pediatric patients in Israel undergoing CT scans in primary care compared to hospitals settings. These cohorts will be further used for evaluating cancer risks.

**Methods:**

The present study was conducted in Schneider Children Medical Center in Israel (SCMCI), the largest tertiary pediatric hospital in the country. Data were collected directly from the listings of the pediatric radiology department for the period 1985–2005. Results were compared with previously published data on pediatric CT in the primary healthcare service performed between 1999 and 2003 in a large health organization, Maccabi Healthcare Services (MHS).

**Results:**

During the study observation periods, 38,351 and 22,223 examinations were documented in 13,726 and 18,075 pediatric patients in SCMCI and MHS, respectively. Compared to pediatric patients in the primary care, patients undergoing CT scans in the hospital were more likely to be younger, to have multiple CT scans, and to be scanned in the trunk. Also, cancer-related indications accounted for nearly 50 % of all CT scans conducted in the hospital compared to only 3 % in primary care settings.

**Conclusions:**

The results indicate major differences in the characteristics of children and adolescents scanned in hospitals compared to primary care settings. Some of these characteristics may be associated with cancer risk later in life, and should be taken into account in cancer risk assessments.

## Background

Over the past three decades, the use of computed tomography (CT) has grown rapidly. In the US, it was estimated that 69 million CT procedures are performed annually in more than 7,500 CT facilities [1] compared to about 3 million in 1980 [2]. The most dramatic increase has been observed among children, who account for 5 % to 11 % of all CT examinations [3–5]. An analysis of the US National Hospital Ambulatory Medical Care Survey indicates that CT use in emergency departments (ED) increased 330 % from 1996 to 2007 [6]. A five-fold increase of CT use was recorded among pediatric patients attending ED between 1995 and 2008 [7]. During the same period, a similar relative increase (from 1.6 to 9.4 per million) was also recorded in the number per capita of CT scanners In Israel [8], one of the world’s leading countries in CT examination rate [5]. According to the United Nations Scientific Commission on the Effects of Atomic Radiation 2000 report, CT scan rate in Israel is 78 per 1000 capita compared to an average of 48 in countries with similar healthcare level [5]. In order to control the use of CT, the number of CT scanners in Israel is closely regulated by the Ministry of Health and current regulations allow only one scanner per 125,000 inhabitants. Thus, Israel still has a relatively small number of CT scanners per capita compared to an average of 20 per million among other countries of the Organization for Economic Co-operation and Development (OECD) [9].

Compared to adults, pediatric patients are at an elevated risk for developing radiation-related cancer [10]. This has been attributed to their developing and rapidly dividing tissues, longer lifetime period at risk for developing cancer and other radiation-related diseases, slimmer body walls and reduced radiation filtering effect, as well as higher radiation exposure from a fixed set of CT parameters in unadjusted machines [11].

The awareness concerning radiation dose and possible cancer risks associated with pediatric CT scans has increased following the publication of several epidemiological studies on this issue that found higher cancer risk with increasing exposure to CT scans. These recently published retrospective cohort studies have estimated long-term cancer risk associated with pediatric CT using data obtained from primary care data [12] or from hospitals [13–15], but not from both sources. They have been criticized for not correcting for potential confounding by predisposing factors known to be associated with increased lifetime cancer risk. The most important overlooked confounders are the medical settings and conditions that prompt the CT scan that is often referred to as ‘confounding by indication’ [16].

To better assess the potential confounding in such observational studies, it is essential to illustrate differences in indications for CT and other characteristics of patient scanned in the community compared to hospitals that may potentially affect lifetime cancer risk. This is particularly important as other cohort studies are underway in number of countries, including the EPI-CT in Europe [17]. Due to its high CT utilization rate and its one of the youngest societies in the Western world [18], Israel provide a unique opportunity to examine patterns of pediatric CT utilization and its potential risks. The objectives of the present analysis were therefore a) to describe the Israeli pediatric CT hospital and community care study cohorts and b) to present important differences between patients undergoing CT scans in community settings and hospitals with regard to potential cancer risk factors. These cohorts are planned to be used in the future to retrospectively assess CT-related cancer risk.

## Methods

To allow the examination of long-term effects and sufficient follow-up time, the Israeli study cohorts were based on pediatric patients who had a CT scan at least ten years ago in community-based or hospital-based settings. The community-based cohort, includes members of a large Israeli health organization (Maccabi Healthcare Services, MHS) scanned between 1999 and 2003 as presented in our earlier study [19]. The hospital-based cohort includes patients scanned in Schneider Children Medical Center in Israel (SCMCI), the largest tertiary pediatric medical center in the country. This 250 bed hospital accounts for 12 % of the total number of pediatric inpatient beds in Israel. It operates seven operating rooms and provides care to 140,000 outpatients annually.

Data were collected directly from the listings of the pediatric radiology department at SCMCI since the introduction of CT to the organization in December 1985 until the end of 2005. We identified all department records from the period prior to the establishment of SCMCI in 1991, when it operated as part of SCMCI. Study data were manually extracted from the paper records by a trained study research assistant (M.L.) and recorded into an excel datasheet. The recorded data included patient unique national identity card number, date of birth, sex, number and types of CT examinations, body site of examination, use of contrast material, and indication for the CT examination. Validation of the paper records of the CT included in the study, was done by searching for an indication of CT scan in the computerized Radiology Information Systems (RIS). For 95 % of CT scans analyzed in this study, an electronic documentation has been found.

CT examinations were described in relation to trends over calendar years with available data, patient ages and sex at examination, frequency of repeated examinations, and region of the body that was scanned. We used four categories of body regions; head, face and neck, trunk (abdomen/pelvis, chest, and spine), and extremities [19]. Comparisons in distribution of body regions between SCMCI and MHS were made for the period 1999–2003 for which data from both organization were available.

The data collected for this study were analyzed using descriptive statistical procedures, including calculating mean and standard deviation (SD) for continuous variables, and frequencies and percentages, along with cross-tabulation chi-squared tests for categorical variables. All statistical analyses were done using a standard statistical software package (IBM SPSS version 18, Chicago, IL, USA). This study protocol was approved by the local review board and did not to require individual patient-level consent.

## Results

During the study observation periods, 38,351 and 22,223 examinations were documented in 13,726 and 18,075 pediatric patients in SCMCI and MHS, respectively. Temporal analysis indicates a rise in the number of CT examinations in SCMCI between the years 1986 (0.3 % of patients, 103 scans) to 1996 (7.5 % of patients, 2946 scans), with an ongoing decline since 1998.

**Fig. 1:**
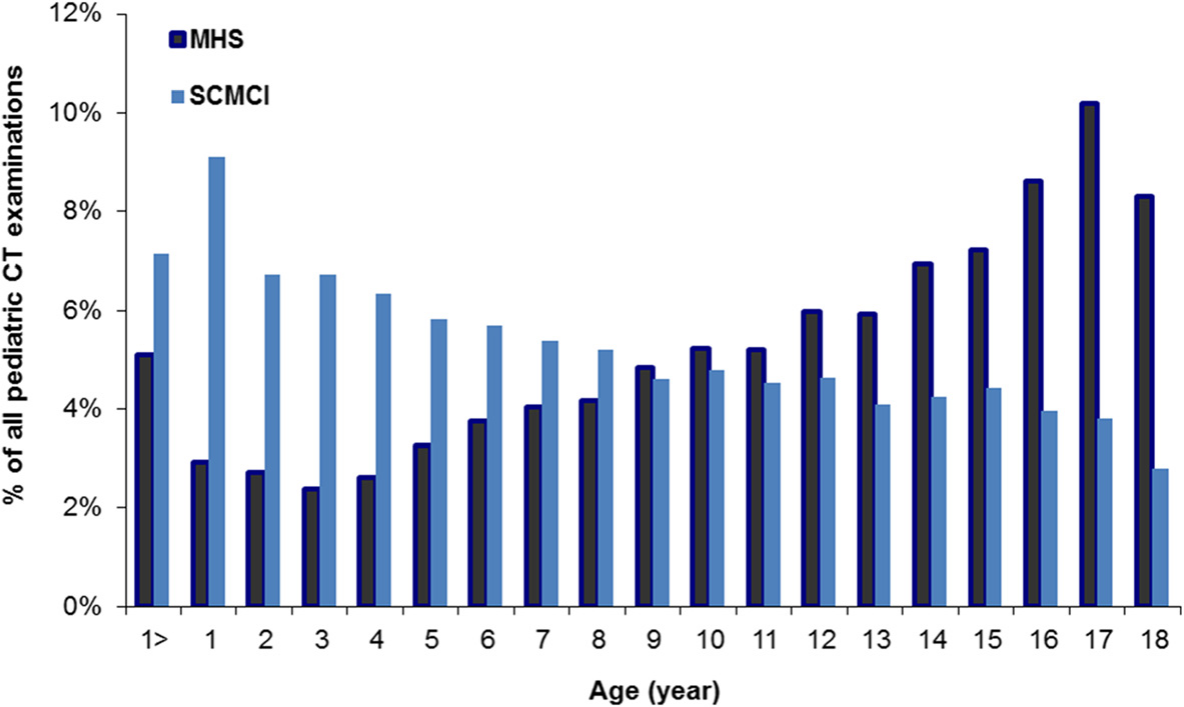
Age distribution of pediatric patients undergoing CT examinations in SCMCI (1985-2005) and previously reported data from primary health care service settings in a large Israeli HMO (1999-2003) [19]

Patients having CT scans in SCMCI had a lower mean age at CT examination (7.7 y, SD = 5.4 y) compared with pediatric patients examined in MHS (mean 11.1 y, SD = 5.5 y). Children in their second year of life accounted for 9 % of pediatrics patients undergoing CT scans in SCMCI, compared to 3 % in MHS (Fig. 1), whereas the opposite pattern was observed at age 18 years where 9 % of MHS patients receiving a CT scan compared to 3 % of SCMCI patients. The number of patients receiving CT scans decreased with age at SCMCI, whereas the number of patients having a CT scan increased with age at MHS. Males accounted for 57 % of all patients undergoing scans in SCMCI and in MHS with little differences across age groups.

The most frequent (37.3 %) indication for CT examination in SCMCI was malignancy or suspected malignancy (Table 1), compared with only 3 % of diagnoses indicating a malignancy in MHS. The proportion of this indication among SCMCI patients increased with increasing age, from 27 % among toddlers aged less than five years to 51 % among examined adolescents (15 y to 18 y). An inverse relationship with increasing age was observed for diseases of the nervous system and sense organs (22.2 % among toddlers compared to 8.7 % among adolescents). Infectious diseases accounted for 27.3 % of scans in MHS, compared to 4.2 % in SCMCI. Diseases of nervous system and sense organs and of trauma, accounted for a similar proportion of all indications or diagnoses in both data sources (approximately 15 % and 10 %, respectively).

**Table 1:** Distribution of indications for pediatric CT, Schneider Children’s Medical Center in Israel 1985–2005, and Maccabi healthcare services (1999–2003)

Indication	% of all CT examination	
	SCMCI	MHS
Cancer	37.30 %	3.30 %
Nervous system	16.80 %	14.40 %
Other/unknown	28.50 %	41.60 %
Trauma	10.00 %	10.10 %
Infectious diseases	4.20 %	27.3 %^a^
Endocrine disorder	3.20 %	3.30 %

^a^Diseases of respiratory or digestive system

**Fig. 2:**
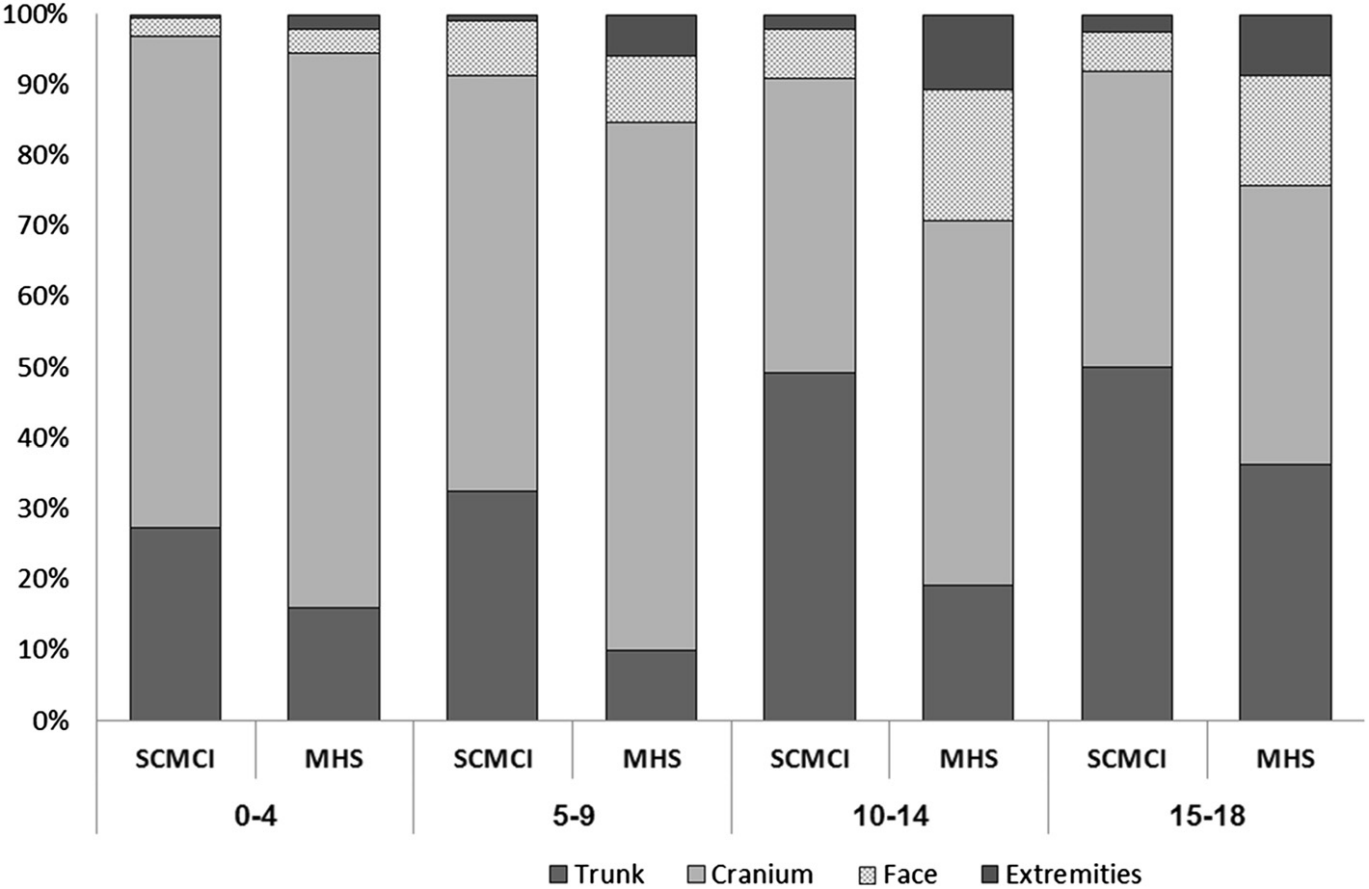
Distribution of CT examinations by body site and patient age at examination in SCMCI and MHS, 1999-2003. (Trunk includes pelvis, spine, chest and abdomen)

In restricting comparisons to a common observation period (1999 to 2003), the cranium was the most frequently scanned body region both in SCMCI (46 %) and MHS (56 %). In all age groups, head CTs accounted for a substantially lower proportion of all scans compared to their proportion in MHS (Fig. 2). Scans of the trunk accounted for 49 % of all CT examinations in SCMCI, compared to only 23 % in MHS. Use of contrast material was documented in 30.3 % of the scans.

Major differences between MHS and SCMCI were found in frequency of repeated CT scans. In the MHS primary health care cohort, 15 % of patients having CT scans had a prior examination within the 5-year period of study, whereas more than half (58 %) of tertiary medical center patients in had a repeated CT examination at the same institution during the follow-up period, and 11.6 % had 15 or more scans (Table 2). The most common diagnosis among patients with repeat exams was cancer or suspected malignancy.

**Table 2:** Distribution of number of CT scans per patient, by age group in SCMCI and MHS

No. of scans	Age group, years								Total	
	<5		5-9		10–14		15–18			
	SCMCI	MHS	SCMCI	MHS	SCMCI	MHS	SCMCI	MHS	SCMCI	MHS
1–4	42.6 %	64.1 %	41.3 %	85.5 %	33.1 %	87.9 %	34.6 %	89.4 %	42.0 %	84.9 %
5–9	24.5 %	33.8 %	25.4 %	13.6 %	20.7 %	11.3 %	25.1 %	10.1 %	24.5 %	14.2 %
10–14	21.9 %	1.7 %	20.7 %	0.7 %	25.4 %	0.7 %	21.4 %	0.4 %	21.9 %	0.7 %
15+	11.0 %	0.4 %	12.6 %	0.2 %	20.7 %	0.1 %	18.9 %	0.1 %	11.6 %	0.1 %
Total	100.0 %	100.0 %	100.0 %	100.0 %	100.0 %	100.0 %	100.0 %	100.0 %	100.0 %	100.0 %

## Discussion

The present report describes the utilization of pediatric CT in the largest pediatric tertiary medical center in Israel over a period of 20 years, since the introduction of this technology into the radiology department. The results indicate major differences between pediatric patients undergoing CT in a tertiary hospital compared to children and adolescents scanned in the primary health care service [19] with respect to risk factors that can be associated with a higher cancer incidence later in life. Firstly, patients scanned in SCMCI were relatively younger compared to those undergoing CT examination in the community. Younger age at exposure is related to increased relative risk for radiation-related cancer risk. It has been shown that the incidence relative risk of cancer associated with CT in children aged 1–4 years is 1.72 (1.44 to 2.05) [20], which is substantially higher than the relative risk of 1.21 among children aged 10 or above. This is of a particular concern in light of the sizeable proportion of individuals in SCMCI cohort (nearly half of the total scanned cohort) that have undergone repeat studies within a period of one year. According to one cohort study, the use of CT scans in children with a cumulative dose of about 50 mGy was associated with a nearly three-fold higher risk of incident leukemia and doses of about 60 mGy almost tripled the risk of brain tumors [13].

Secondly, a much greater proportion (50 %) of the SCMCI patients were scanned due to an indication of malignancy or cancer-related conditions, compared to only 3 % in primary health care settings. History of cancer or precancerous condition in children is associated with an increased risk of second malignancies [21]. Therefore, hospital-based cohorts of pediatric patients undergoing CT examination are at higher risk of cancer development compared to primary care cohorts. Third, an additional potential for increased cancer risk in our cohort arises from the distribution of scanned body regions. The present study population was characterized by a larger proportion of trunk scans that accounted for nearly half of all CT examinations. Trunk CT scans have been associated with a higher lifetime attributable risks of solid cancer compared to head CT [20]. Future analyses should therefore take into account these potential confounders and biases in estimating cancer risk and may benefit from assessing adjusted cancer risk rates of unexposed individuals as well as conducting sensitivity analyses for various indications for CT examinations.

While studies from the UK [22] and the US [23] indicate that the use of pediatric CT has been growing during mid-1990’s and early 2000’s, a more updated data indicates a steady decline in CT utilization as a proportion of all imaging studies in pediatric facilities across North America [24]. Similarly, we found a gradual decrement in number of CT scans performed since 1998 in the SCMCI. While the growth of use has been previously explained by a lower threshold for ordering these studies in routine clinical practice, the decline in use since late 1990’s is probably due to the introduction of new devices to SCMCI that do not involve ionizing radiation, including magnetic resonance imaging (MRI), that has been increasingly used at SCMCI since 1995 similar to pediatric facilities in the US [24]. During this period, the use of MRI in the general population of Israel has increased from 6 per 1000 in 1995 to 10 per 1000 in 2005 [25].

Similar to recently published studies [12, 22, 23], imaging of the cranium was one of the most common organs scanned both in SCMCI and in MHS, particularly among young children. One of the main reasons for conducting pediatric CT scans is headache [26]. Although neuroimaging is considered unnecessary in the diagnosis of headache when no other neurologic symptoms are present to rule out serious intracranial pathology, a recently published retrospective research study reported that 26 % of the children with medical claims for headache underwent CT scan, 75 % of them within 1 month of index diagnoses [27]. These results underline the need for increased awareness regarding the appropriate use of these procedures and balancing the long-term risks inherent in radiation exposure with the necessity for making clinical decisions.

The risks of radiation exposure in children are also not restricted to the development of cancer. For example, repeated head CT that includes imaging of the lens of the eye may increase the risk of later cataract formation [28]. The orbits comprised 3 % of the pediatrics CT scans in SCMCI. New studies have suggested an elevated risk for the development of cataract in populations exposed to doses of ionizing radiation well below the previous reported threshold for radiation cataract, which was 2.0 Gy [28]. These findings reinforce the importance of judicious use of imaging procedures that utilize ionizing radiation, particularly in children.

There are several strengths that should be pointed out in our research. The first is a large sample size, due to the documentation of all the CT scans in SCMCI over 20 years, since the introduction of CT to the organization. Secondly, the initial collection of data took place in the radiology department, with the full cooperation of the department head and data were abstracted from the original examinations books. Radiologists had recorded the CT exam data in the books, ensuring that we had a good quality data. Several study limitations should be discussed. The first is selection bias. SCMCI is a tertiary children’s hospital and therefore receives more sick and complicated pediatric patients than seen in primary medical facilities. However, because of those differences, the characterization of the CT usage pattern and comparison between the two facilities is important for the more accurate understanding of the pediatrics CT usage in Israel. In addition, some of the pediatric patients could have been scanned in the referring hospitals and not document in the current study. Therefore, our assessments of repeated CT examinations are probably underestimated.

The utilization rate of pediatric CT in our study peaked in the late 1900s and early 2000s, and seems to be dropping since. The increased public awareness to cancer risks associated with CT scans and the need for patient-specific CT examinations in young patients, has rowsep="1"resulted in the Image Gently campaign, targeting towards images that provides diagnostic image quality at the lowest possible radiation dose (As Low As Reasonably Achievable, or ALARA) [29]. Implementation of the ALARA principle includes adjustments of pediatric scan protocols for patient size and careful planning the set of scan protocols according to patient’s weight intervals, scan body region, and indication.

## Conclusions

In this study, several main differences were found between children scanned in a tertiary pediatric hospital versus primary health care services. Those differences (younger age at exposure, higher rate of multiple CT exams, and increased prevalence of trunk radiation) have been found to be related to a higher risk for developing cancer [20]. Health-care providers need to be aware of those at the highest risk for radiation-related cancer to avoid unnecessary examinations. Because doses from CT examination are directly related to the quantity of radiation exposure and therefore to the cancer risk [11], minimizing CT examinations and lowering radiation dose parameters to as low as reasonably possible, can lower that risk. A careful consideration of the medical benefits and radiation-related harms of using CT for pediatric patients requires empirical data on the long term development of cancer and other health effects among these patients. As part of an international effort to quantify the radiation-related risks of pediatric CT, the patient listings of the two cohorts are currently cross-linked with the Israeli Cancer Registry to detect incident cancer cases and to calculate long-term cancer risks. The results of the international study will be used in order to develop strategies to improve patient safety.

## Abbreviations

CT: Computed tomography
ED: Emergency departments
OECD: Organization for Economic Co-operation and Development
RIS: Radiology Information Systems
SD: Standard deviation
SMN: Secondary malignant neoplasm
